# miRNA-1183-targeted regulation of *Bcl-*2 contributes to the pathogenesis of rheumatic heart disease

**DOI:** 10.1042/BSR20201573

**Published:** 2020-11-02

**Authors:** Ni Li, Linwen Zhu, Hua Zhou, Dawei Zheng, Guodong Xu, Lebo Sun, Jianqing Gao, Guofeng Shao

**Affiliations:** 1 Department of Cardiothoracic Surgery, Ningbo Medical Centre Lihuili Hospital, Ningbo University, Ningbo, Zhejiang 315041, China; 2 Institute of Pharmaceutics, College of Pharmaceutical Sciences, Zhejiang University, Hangzhou, China

**Keywords:** apoptosis, B cell lymphoma-2 gene, miRNA-1183, rheumatic heart disease, target gene prediction

## Abstract

To determine whether up-regulation of miR-1183 targeting the gene for anti-apoptotic factor, B-cell lymphoma 2 (BCL-2) contributes to apoptosis in patients with rheumatic heart disease (RHD). Peripheral blood samples were isolated for miR-1183 characterization. The function of miRNA-1183 in RHD using miRNA mimic on PBMCs and THP-1 cell models. The binding of miR-1183 and *Bcl-2* gene was confirmed by luciferase activity test. We also measured expression levels of BCL-2 in heart valve tissue from patients with RHD using ELISA and immunohistochemistry. *In silico* analysis and reporter gene assays indicated that miR-1183 directly targets the mRNA encoding BCL-2. It is found that miR-1183 binds directly to the 3′UTR of the BCL-2 mRNA and down-regulates the mRNA and protein levels of BCL-2. Overexpression of miR-1183 in RHD patients and cell lines down-regulated BCL-2 expression and induced apoptosis. With the progression of the disease, the expression of BCL-2 in the heart valve tissue of patients with RHD decreased. MiRNA-1183 is up-regulated in RHD and induces cardiac myocyte apoptosis through direct targeting and suppression of BCL-2, both of which might play important roles in RHD pathogenesis. During the compensatory period of RHD, up-regulated miR-1183 destroyed the balance of apoptosis proteins (Bax and BAK) in *Bcl-2* family, enhance the apoptosis cascade reaction and reduce the anti apoptosis effect. The significantly higher expression levels of miR-1183 appear to play distinct roles in RHD pathogenesis by regulation BCL-2, possibly affecting myocardial apoptosis and remodeling in the context of RHD.

## Introduction

Rheumatic heart disease (RHD) is an autoimmune disease induced by group A hemolytic streptococcus infections, primarily affecting people under 40 years old, and often damaging endocardium, pericardium, and myocardium [1,2]. Many studies have confirmed that the pathogenesis of RHD is related to immune and autoimmune responses after viral or bacterial infections; nevertheless, the pathogenesis of its pathogenesis remains unclear [3]. *Streptococcus* cross-reactions act on heart tissue, driving CD4^+^ T cells to expand and produce autoimmune damage, which is considered to be the most likely pathogenesis of RHD [4,5]. Rheumatic carditis can involve the endocardium, myocardium, and pericardium. It can occur repeatedly and acutely during the course of the disease, causing valve congestion, swelling, fibrosis, and adhesion of tendon papillary muscle. All these effects produce permanent pathological changes in the myocardium after becoming chronic, which is a characteristic feature of heart failure in RHD patients [6]. The anti-apoptotic factor, B-cell lymphoma 2 (BCL-2), was found to be decreased in RHD patients. The accumulating data indicate likely undergo apoptotic processes.

At present, the most effective and direct treatment of RHD is valve replacement surgery; however, most patients need to take warfarin anticoagulation for a long time after surgery, and their quality of life is significantly reduced. RHD involves complex pathological changes. MicroRNA is a hot topic in the field of cardiovascular research in recent years [7–9]. Nevertheless, there are few reports of microRNA in the research of RHD [10,11].

Many studies have reported that microRNA is involved in myocardial fibrosis. It was shown that miRNA directly inhibited the expression of various extracellular matrix proteins, and it regulated a variety of signal pathways related to fibrosis, especially fibrosis occurring after necrosis caused by myocardial infarction [12–14]. At present, there is little research on the role of miRNA in RHD, and the molecular biology of RHD may prove fruitful. Molecular techniques can detect RHD early, allowing for early treatment. It is hoped that, in the near future, miRNA will become a tool for early diagnosis of RHD. Furthermore, it may provide new ways to treat valvular fibrosis. Nevertheless, the molecular mechanisms through which miRNAs influence myocardial apoptosis and remodeling of RHD have not yet been fully elucidated.

Therefore, in the present study, we focused on the functions of miR-1183 in RHD models, specifically those inducing apoptosis. BCL-2 might act as a target gene of miR-16-5p. Here, we investigated whether levels of miR-1183 were altered in patients with RHD, as well as in primary human peripheral blood mononuclear cells (PBMCs) and human myeloid leukemia monocyte (THP-1) cells. The aim was to determine whether alteration of BCL-2 expression levels might contribute to the pathophysiology of RHD.

## Methods

### Experimental specimen and clinical characteristics

From March 2017 to December 2019, we enrolled 20 patients with RHD and 20 healthy controls without medical problems. All experiments were carried out in accordance with relevant guidelines and regulations. The inclusion criteria of the RHD group were as follows: (i) RHD diagnosed by echocardiography, including diagnosis of mitral valve prolapse because of mitral chordae tendineae fracture and mitral insufficiency, scheduled for mitral valve replacement, aortic insufficiency scheduled for aortic valve replacement, or tricuspid regurgitation or insufficiency of closure scheduled for valve replacement or valvuloplasty; and (ii) left ventricular ejection fraction (EF) >50%. Pulmonary arterial pressure was measured by echocardiography and divided into low, mild and severe conditions: low PAH (PASP < 30 mmHg), mild PAH (30 mm Hg ≤ PASP < 55 mmHg), and severe PAH (PASP ≥ 55 mmHg). Exclusion criteria were as follows: valve damage caused by congenital, syphilitic heart valve disease; infective endocarditis; papillary muscle dysfunction; tendon rupture; myxoid degeneration; and other autoimmune diseases. The characteristics of the cases and controls are shown in Table 1.

**Table 1:** Clinical characteristics of patients with RHD and normal controls

	RHD group	Healthy controls
Blood samples	20	20
Sex (Male/Female)	7:13	10:10
Age	56.7 ± 6.0	49.9 ± 6.2
Smoking	5(25%)	4(20%)
Hypertension	5(25%)	0
Diabetes	0	0
Hereditary diseases	0	0
Immune diseases	0	0
Tumor	0	0
Other vascular diseases	0	0
Trauma	0	0
Gestation	0	0
Cardiac function grade (NYHA grade)	II∼IV	/
EF(%)	59.22 ± 7.14	/
LVEDD (mm)	48.65 ± 4.48	/
PASP (mmHg)	51.1 ± 4.96	/

Notes: LVEDD, left ventricular end-diastolic size; EF, ejection fraction. Cardiac function grades: NYHA divided into Grade II, Grade III, Grade IV. PASP, pulmonary artery pressure.

In the healthy control group, blood lipid levels, blood pressure, and blood glucose were all within normal ranges, and heart function and pulmonary artery pressures were normal; no heart disease was detected. We collected 5 ml of venous blood from the normal and case groups into tubes containing EDTA. After centrifugation at 4°C for 15 min, the supernatants were removed and stored at −80°C. We obtained the approval of the medical ethics committee of Li Huili Hospital, and written informed consent was obtained from all patients.

### RNA extraction

The reverse transcription kit and real-time PCR kit were purchased from TaqMan and assays were performed according to the manufacturer’s instructions. Using the ultramicro spectrophotometer k5600, 1 μl RNA sample was used for determination. We recorded the concentration and purity of each sample. The ratio of absorbance at 260 nm to that at 280 nm was between 1.8 and 2.0, which can be used in gene chip detection and PCR.

### Construction of the miR-1183 expression vector and the target gene 3′UTR double luciferase expression vector, transfection and luciferase assays

Plasmid transfections for luciferase assays in 293T cells were performed with 3′UTR luciferase 0.1 μg, miRNA expression plasmid 0.4 μg, Renilla plasmid 0.02 μg in a 24-well plate as described by the manufacturer. The binding between miRNA and target gene 3′UTR was verified using double fluorescent enzyme reporter gene technology. Luciferase activity was measured 48 h post transfection using the Dual Luciferase Reporter Assay System as described by the manufacturer (Promega).

The names of plasmids in the detection system were as follows: Mir1183-NC: microRNA empty plasmid, as the negative control of the target microRNA plasmid; Mir-1183: miRNA vector plasmid, expressing the target microRNA (hsa-mir-1183 (21031-1)); 3′UTR-NC: 3′UTR empty plasmid as negative control of target gene 3′UTR plasmid; 3′UTR: target gene 3′UTR plasmid (BCL2 (21010-1)); 3′UTR-MU: target gene 3′UTR mutant plasmid (BCL2 (21011-1)); posotive control 3′UTR: TRAF6 gene 3′UTR plasmid; Vector plasmid of posotive control miRNA: hsa-mir-146b. Hsa-miR-1183 mature miRNA sequence is CACUGUAGGUGAUGGUGAGAGUGGGCA. The control RNU6b sequence is CGCAAGGATGACACGCAAATTCGTGAAGCGTTCCATATTTTT.

### Isolation, culture, and identification of primary cells

Peripheral blood mononuclear cells (PBMCs) were separated using Ficoll density gradient centrifugation. The peripheral blood samples and d-Hanks solutions were collected and diluted at 1:1. The volume ratio of diluted blood to lymphocytes was 1:1. Samples were centrifuged at 2000 rpm/min at room temperature for 20 min. After centrifugation, the fluid was divided into four layers. The monocytes in the cloud layer were removed using a capillary dropper, and then centrifuged repeatedly by adding d-Hanks solution to prepare monocyte suspensions. The cell precipitates were cultured in RPMI 1640 complete medium, and cultured for 24 h. We added macrophage colony stimulating factor to continue the culture. The medium was changed every 72 h and RPMI 1640 containing 10 ng/ml M-CSF + 10% fetal bovine serum was added. The subsequent experiment was carried out 7 days later.

### Human peripheral blood mononuclear (THP-1) cells

THP-1 cells were cultured in RPMI 1640 medium containing 10% FBS and double antibodies. Macrophages were induced by propylene glycol methyl ether acetate (PMA). After 3 days of continuous culture, the cells were replaced by 1640 cells without PMA.

### Cell transfection, RT-PCR

Lipo2000 was used as the transfection vector (50 μl + 2500 μl opti) in the process of cell transfection. Mimic-1183, control group (40 μl + 1000 μl opti), 100 μl per well, mixing for 5 min, standing for 20 min. After 48 h of transfection, the supernatants were removed, some of the extracted RNA was used for RT-PCR follow-up experiments. The cDNA was prepared using the TaqMan miRNA qRT PCR kit as previous study.

### Measurement of BCL-2 expression in heart valve tissue and plasma using ELISA

We added 300 μl washing solution and soaked it for 30 s. We then added 100 μl 2× diluted standard into the standard well. We added 100 μl standard diluent or culture medium into the blank wells. We then added 80 μl × detection buffer and 20 μl serum sample into the sample wells. Cell culture supernatant: we added 100 μl cell culture supernatant into sample wells, and added 50 μl 1:100 diluted BCL-2 antibody to each well. The plates were sealed and incubated at room temperature for 2 h, after which they were washed six times. We added 100 μl of chromogenic substrate into each well, in the dark, and incubated at room temperature for 5–30 min. Finally, we added 100 μl of termination solution to each well. Within 30 min, the optical density was measured at 450 nm.

### Immunohistochemistry (paraffin section IHC)

Sections were baked for 40 min, and placed into the reagent tank for dewaxing, hydration, and incubation with 3% hydrogen peroxide (diluted with pure water) for 10 min to eliminate the activity of endogenous peroxidase. Paraffin-sections were then deparaffinized and rehydrated. Antigen retrieval was performed using heat-incubation in epitope retrieval solution (IHC World, U.S.A.) for 30 min. The primary antibodies used for immunohistochemistry were anti-bcl-2 (1:200).

### He staining of tissue sections

After baking, the above tissue sections were successively infiltrated into xylene 1 and dewaxed with xylene II for 10 min. Immerse in anhydrous ethanol I, anhydrous ethanol II, 95% alcohol, 80% alcohol, and 70% alcohol for 2 min respectively. Hematoxylin was dyed for 3 min, then immersed in 50%, 70% and 80% alcohol for 2 min respectively, then immersed in eosin for 5 s. Mirror inspection after neutral gum sealing.

### Statistical analysis

For pyrosequencing, DNA quality was assessed using GraphPad Prism 6 analyzer software. The Student’s *t*-test and ANOVA were used to test for differences in methylation levels for categorical variables. For qRT-PCR results, we used the paired *t*-test or the χ^2^ test as appropriate. Data are shown as means ± SD. *P*<0.05 was regarded as significant.

## Results

### MiR-1183 down-regulates expression of the anti-apoptotic factor BCL-2

**Figure 1:**
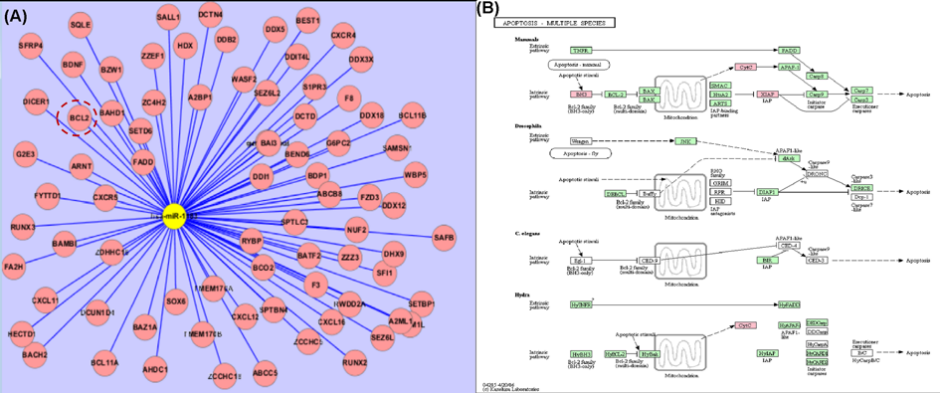
has-mir-1183 target gene prediction (**A**) Miranda, miRBase and targetscan jointly predict target genes (target gene bcl-2, cxcr4, etc). (**B**) KEGG analysis on apoptosis pathway.

In the present study, we used 40 peripheral blood samples for RNA extraction. Clinical information is displayed in Table 1. On the basis of our previous work, we used the GeneChip® miRNA Array to profile the quantitative change of miRNAs in plasma of 1, 2, N3 (control group) and 4, 5, 6 RHD patients, differently expressed miRNAs were screened on the chip (Table 2). We found that the expression of miR-1183 in the RHD group was 16.5 times higher than that of the healthy control group (*P*<0.05). The high expression levels of miR-1183 in plasma was verified using PCR (with the miR-1183 Ct value 33.75 compared with control group Ct value 36.24). In the detected the expression level of miR-1183 using qPCR, MiR-1183 is up-regulated in the blood samples from RHD patients; meanwhile, the *Bcl-2* mRNA was significantly down-regulated in RHD cases compared with the healthy normal control samples. However, when detecting the correlation between miR-1183 and BCL-2, the *P* value is 0.2143. The significance was not obvious (shown in Supplementary Figure S1). To investigate the underlying mechanisms, we searched Miranda, miRBase, and TargetScan for targets of miR-1183 genes, with the well-known anti-apoptotic factor BCL-2, showing its prediction probability. Among them, BCL-2 and other apoptosis-related targets *bcl*11A, *bcl*11B were also found (Figure 1A). According to the prediction of target genes and the results of cell pathway involved, combined with a literature search for the pathogenesis of RHD, we studied whether miR-1183 is involved in the signal pathway of apoptosis related genes. KEGG analysis showed that the effect of miR-1183 and BCL-2 on the apoptosis pathway (Figure 1B). Through KEGG analysis, the possible mechanism of apoptosis related pathway is to promote/inhibit the balance of apoptosis proteins (Bax, BAK) in *Bcl-2* family, promote the release of cytochrome *c*, enhance the apoptosis cascade reaction and reduce the anti apoptosis effect. Agarose gel electrophoresis and sequencing verified that the luciferase expression vector was successfully constructed. Binding between miRNA and the target gene 3′UTR was verified using a double fluorescent enzyme reporter assay. These results suggest that miR-1183 is upregulated and suppresses the expression of BCL-2 in these cells.

**Table 2:** quantitative analysis and quality assurance of 6 samples for miRNA gene chip

Group	No.	OD260/280	OD260/230	concentration (ng/μl)	Capacity (μl)	Quality (ng)	Up-regulation of gene chip detection
Control group	1	1.74	1.87	28.93	10	289.30	0.13
	2	1.79	1.88	21.69	10	216.90	0.11
	N3	1.80	1.91	29.88	10	298.80	0.08
RHD group	4	1.71	1.96	26.19	10	261.90	2.5
	5	1.73	1.86	24.33	10	243.30	1.36
	6	1.79	1.80	23.23	10	232.30	1.36

### The binding of miR-1183 and Bcl-2 gene was confirmed by luciferase activity test

**Figure 2:**
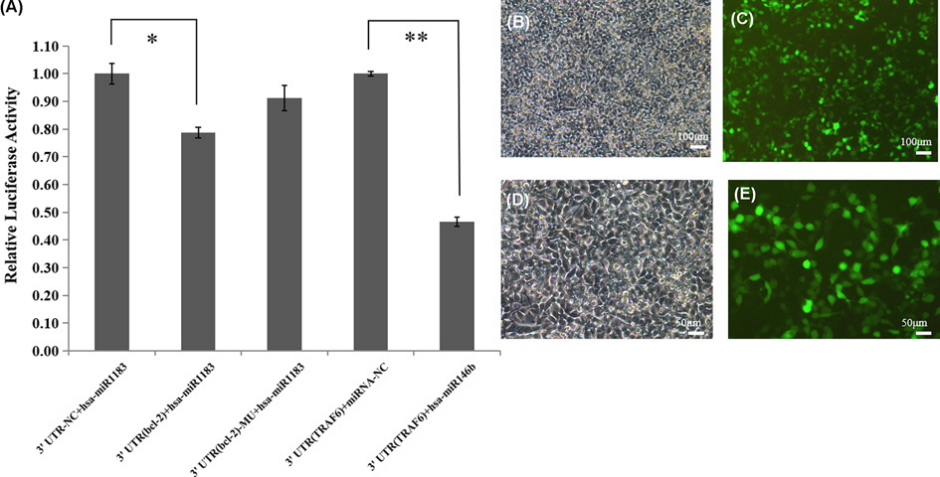
The binding of mir-1183 to *Bcl-2* by the method of double luciferase reporter gene (**A**) Detection of 3′UTR reporter gene (luciferase). (**B–E**) Fluorescent photos of GFP plasmid transfected. (B) GFP 100× B; (C) GFP 100× G; (D) GFP 200× B; (E) GFP 200× G (*P<0.05, **P<0.01).

To examine whether miR-1183 directly targets BCL-2, we further investigated the binding of miR-1183 to Bcl-2 using the double luciferase reporter assay (Figure 2). In our 3′UTR reporter gene system, the 3′UTR region of the target gene is constructed behind the reporter gene luciferase. By comparing the overexpressed microRNA, the change of reporter gene expression (monitoring the change of luciferase activity) can quantitatively reflect the inhibition of microRNA on the target gene. Compared with group (3′UTR+ hsa-mir1183) and group (3′UTR (Bcl-2) + hsa-mir1183), luciferase expression decreased by *t*-test analysis (*P*<0.05). This is the same trend as the positive reference TRAF6 and hsa-mir146b that indicated that mir-1183 combined with target gene 3′UTR and inhibited its expression (Figure 2A). The fluorescent photos of GFP plasmid transfected in the same batch are as shown in the Figure 2B–D.

### Gain-of-function miRNA mimic

**Figure 3:**
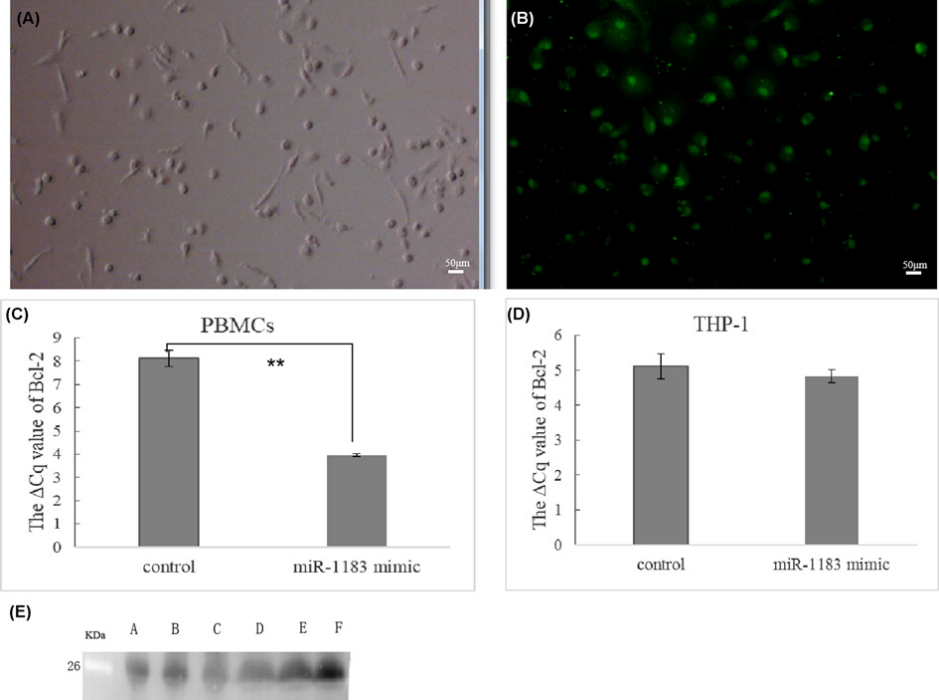
Functional acquisition study of miR-1183 on primary peripheral blood mononuclear cells (PBMCs) and THP-1 cells (**A** and **B**) Effect of mir-1183 transfection by primary peripheral blood mononuclear cells (PBMCs); (A) under ordinary microscope and (B) under fluorescence microscope. (**C** and **D**) Expression of *Bcl-2* mRNA in PBMCs and THP-1 cells transfected with mir-1183 mimic. (**C**) PBMCs *Bcl-2* mRNA expression by PCR; (**D**) THP-1 *Bcl-2* mRNA expression by PCR (**P<0.01). (**E**) Western blot for the detection the expression of Bcl-2 in RHD patients with high expression of mir-1183. (A**–**C) Three cases of RHD groups; (D–F) Three control groups (scale bar: 50 μm).

Because the development of RHD is closely related to immunity, we selected PBMCs and THP-1 cells as the main research objects, and studied synthetic genes associated with specific miRNA mimics focused on the level of mRNAs, study on the interaction between miR-1183 and target gene BCL-2. After 48 h of transfection of PBMCs with FAM-labeled miRNA negative control and FAM-labeled miRNA inhibitors (negative control), the expression of green fluorescence in the cytoplasm of PBMCs was observed under fluorescence microscopy. This showed that the transfection was successful (Figure 3A,B).

The transfection results were further verified using real-time fluorescent quantitative PCR. When we examined BCL-2 mRNA levels in PBMCs cells and THP-1, we found that levels of BCL-2 mRNA were down-regulated by the miR-1183 mimic. As shown in Figure 3C,D, expression levels of BCL-2 in PBMCs and THP-1 cells transfected with miR-1183 mimics were measured. Compared with the normal control group, the expression of BCL-2 A1 in the transfected group was decreased (as shown by the ⊿CT value, with GAPDH as the internal reference), and expression levels of the miR-1183 inhibitor group were greater than those of the control group. From Western Blot result, miR-1183 down-regulates BCL-2 at mRNA levels by the detection the expression of Bcl-2 in RHD patients with high expression of miR-1183 (Figure 3E). It was also turned out that in the functional analysis of H9c2 cells, flow cytometry was used to detect the apoptotic ability. Knockdown of miR-1183 could inhibit the apoptosis of H9c2 cells, and overexpression of miR-1183 could promote the apoptosis of H9c2 cells (shown in Supplementary Figure S2). The anti-apoptotic factor, BCL-2 was proved to be related to the expression of miR-1183, it is speculated that abnormal expression of miR-1183 might be related to apoptosis.

### Measurement of BCL-2 expression in heart valve and serum of RHD patients

**Figure 4:**
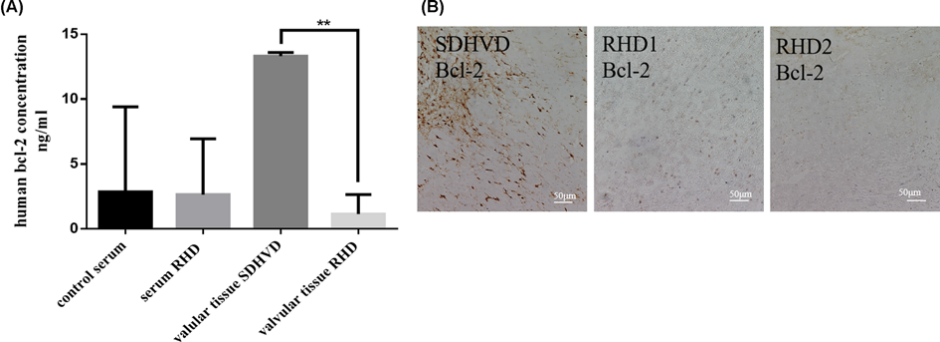
Detection of *Bcl-2* on heart valve and serum in patients with rheumatic heart disease (**A**) ELISA determination. (**B**) Comparison of the expression of *Bcl-2* between one case of mild degenerative heart disease and two cases of rheumatic heart disease by immunohistochemistry (scale bar: 50 μm) (**P<0.01).

To further elucidate the relationship between expression of BCL-2 and the progression of RHD, we found that the expression of BCL-2 protein in the heart valves tissue of rheumatic heart disease patients was lower than that of mild degenerative heart disease patients (SDHVD) (Figure 4A). It is also demonstrated that the expression of *Bcl-2* in two rheumatic heart disease patients was significantly lower than that in the control group by immunohistochemistry (Figure 4B). This may be related to the decrease of *Bcl-2* in immunohistochemistry.

## Discussion

MicroRNA has become a research hot spot in the field of tumor and cardiovascular in recent years. It has been reported that microRNAs are involved cardiovascular diseases such as coronary heart disease and myocardial fibrosis. MiRNA directly inhibits the expression of a variety of extracellular matrix proteins, and it also regulates a variety of signal pathways related to fibrosis, especially myocardial infarction-induced fibrosis. There are several reports on miRNA in serum, and the study of circulating miRNA has become an intense area of research. There are also several reports of the abnormal expression of circulating miRNA in various diseases. These molecules can serve as molecular biological markers for the early diagnosis of various clinical diseases. It was reported that some miRNAs are specifically expressed in heart tissue and pulmonary hypertension [15,16]. Nevertheless, no specific expression of miRNAs such as miR-208 have been found in other tissues. When myocardial cells are injured, these specific miRNAs are released into circulating blood, suggesting that miRNAs can be serve as markers of myocardial injury. Nevertheless, no miRNA has been studied with respect to its role in RHD.

The pathological changes of RHD occur in the valves, primarily manifesting as fibrosis and hyperplasia. In our gene chip analysis, more than 450 miRNAs showed up-regulation or down-regulation in varying degrees in patients with RHD and pulmonary hypertension. The combined fluorescence quantitative PCR experiment confirmed the possibility of the relationship between miR-1183 and RHD. On the basis of our previous study, we found that miR-1183 was highly expressed in the blood of patients with RHD. Circulating RNA is derived from apoptosis or necrosis of cells. The combination of miRNA with various proteins protects them from degradation in circulation. To further explore the mechanism of high expression microRNA-1183 in plasma, we used Miranda, miRBase, and TargetScan to predict the target genes of miR-1183. Taken together, the present and previous findings suggest that increased miR-1183 expression promotes apoptosis, thereby becoming involved in RHD.

We then sought to identify apoptosis-related target genes of miR-1183. Hundreds of target genes including BCL-2, EGFR, and CXCR4 were found. The target genes *bcl*2A1, EGFR, and Bax were found by miR-1183 on target gene prediction software. We found that apoptosis was involved in the pathogenesis of RHD. The BCL*-*2 gene was closely related to the pathological changes of RHD. The relationship between miR-1183 and related target genes was studied by functional acquisition and functional deletion. The expression levels of *bcl2*A1 gene transfected with miR-1183 in human peripheral blood mononuclear cells decreased significantly. Combined with RT-PCR experiments, these results suggest that apoptosis is involved in the pathogenesis of RHD, and the *bcl*-2 gene was involved in the regulation of apoptosis [17–19]. During the compensatory period of RHD, high expression levels of miR-1183 trigger decrease in *bcl*-2 gene expression levels, thereby regulating apoptosis. For further study, we selected BCL-2, which is an anti-apoptotic protein that prolongs cell survival. Aberrant down-regulation of BCL-2 disrupts mitochondrial membrane integrity, induces the mitochondrial release of pro-apoptotic proteins, and triggers caspase activation, and cytoskeletal degradation, resulting in apoptosis. In the present study, we found that miR-1183 binds directly to the 3′UTR of the BCL-2 mRNA and down-regulates the mRNA and protein levels of BCL-2 in 293T cells. These findings suggest that BCL-2 plays an important role in regulation of apoptosis in RHD.

We speculate that the mechanism might be, up-regulated miR-1183 promote/inhibit the balance of apoptosis proteins (Bax, BAK) in *Bcl-2* family, promote the release of cytochrome *c*, enhance the apoptosis cascade reaction, and reduce the anti apoptosis effect. The up-regulation of mir-1183 may affect the expression of *Bcl-2* and the level of cardiomyocyte apoptosis, which may be one of the reasons for the further development of rheumatic heart disease.

The purpose of the present study was to explore the application of differentially expressed miRNAs as biomarkers in the diagnosis of RHD, and to help elucidate the mechanisms. As a preliminary study, we used real-time quantitative PCR to measure CT value differences of miR-1183 and verified the results using a gene chip assay. To date, there are few reports of miRNA in RHD. MiR-1183 screened by microarray has not been found to be related to the cardiovascular system.

There are still limitations in the present study. The sample size needs to be further expanded: only 40 samples were selected for PCR verification, which is far from sufficient. There is no gold standard clinical diagnosis of RHD. Rheumatic antibody cannot diagnose patients with RHD in the inactive period; the possibility of RHD can only be tested using color Doppler echocardiography and medical history. However, combined with relevant research involving miRNA, we can find that the specific expression of miRNA in patients with RHD is due to the hyperplasia and fibrosis of heart valve, which leads to the increase of miRNA expression in patients with rheumatic heart disease. This prediction requires experimental verification. No primary cardiomyocytes were cultured from patients with rheumatic heart disease,this should be a better cell model, but due to the limited conditions, the cells could not be effectively analyzed. In the present study, we selected PBMCs [20,21] and THP-1 cells [22] and human miRNA mimics for functional acquisition and miRNA inhibitors for functional deletion. We focused on mRNAs, simulating the role of miRNA *in vivo*, to determine whether the progression of RHD was related to immunity; nevertheless, there are some defects in design. It is necessary to carry out further biological function analysis to determine the pathophysiological mechanisms of these miRNAs in RHD.

## Conclusions

In conclusion, we demonstrated that miR-1183 is differentially expressed in RHD. The significantly higher expression levels of miR-1183 appears to play a role in pathogenesis RHD by regulation of the anti-apoptotic protein, BCL-2, which might affect myocardial apoptosis and remodeling in RHD.

## Supplementary Material

Supplementary Figures S1-S3

## Data Availability

All data included in the present study are available upon request by contact with the corresponding author.

## Abbreviations

*bcl-*2: B-cell lymphoma 2
miR-1183: hsa-microRNA-1183
PBMC: human peripheral blood mononuclear cell
RHD: rheumatic heart disease
SDHVD: mild degenerative heart disease
THP-1: human myeloid leukemia monocyte cell
