# Supplementary material for: miRNA-1183-targeted regulation of Bcl-2 contributes to the pathogenesis of rheumatic heart disease
Source: Biosci Rep. 2020 Nov 2;40(11):BSR20201573. doi: 10.1042/BSR20201573 (PMC7607189; doi:10.1042/BSR20201573)
Supplement: Supplementary Figures S1-S3 [file BSR-2020-1573_supp.pdf]

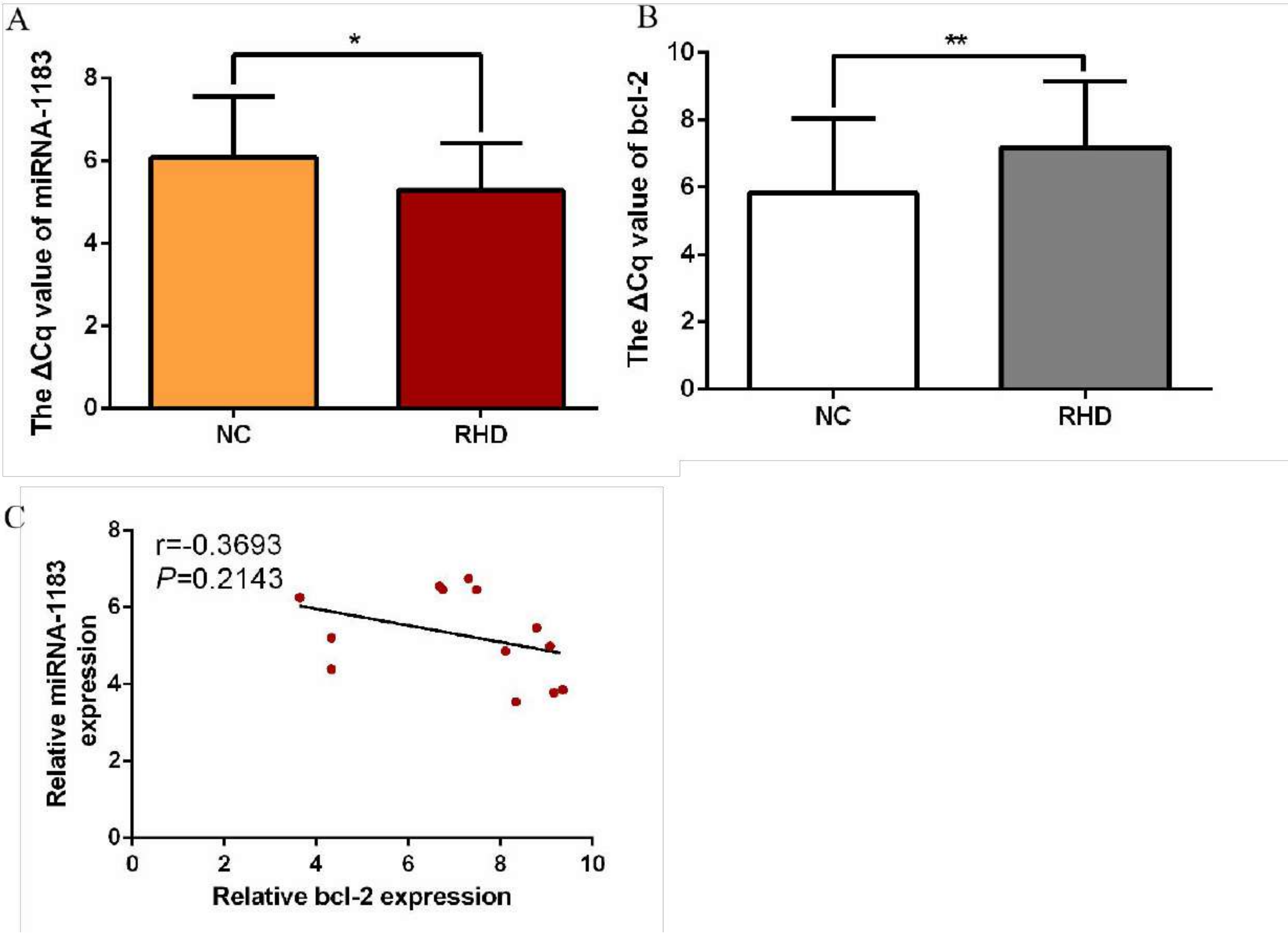

A
*
The ΔCq value of miRNA-1183
0
2
4
6
8
NC
RHD
B
**
The ΔCq value of bcl-2
0
2
4
6
8
10
NC
RHD
C
r=-0.3693
P=0.2143
Relative miRNA-1183 expression
0
2
4
6
8
0
2
4
6
8
10
Relative bcl-2 expression

Supplementary Figure 1. MiRNA-1183 and bcl-2 mRNA expression levels in RHD cases and healthy normal control samples. (A) The expression levels of miRNA-1183 expression in RHD cases were significantly lower than healthy normal control samples (n = 13)，*P < 0.05. (B) The expression levels of bcl-2 mRNA was significantly downregulated in RHD cases compared with the healthy normal control samples,**P <0.01. (C)Correlation between miR-1183 and BCL-2. Higher ΔCq value means lower expression.

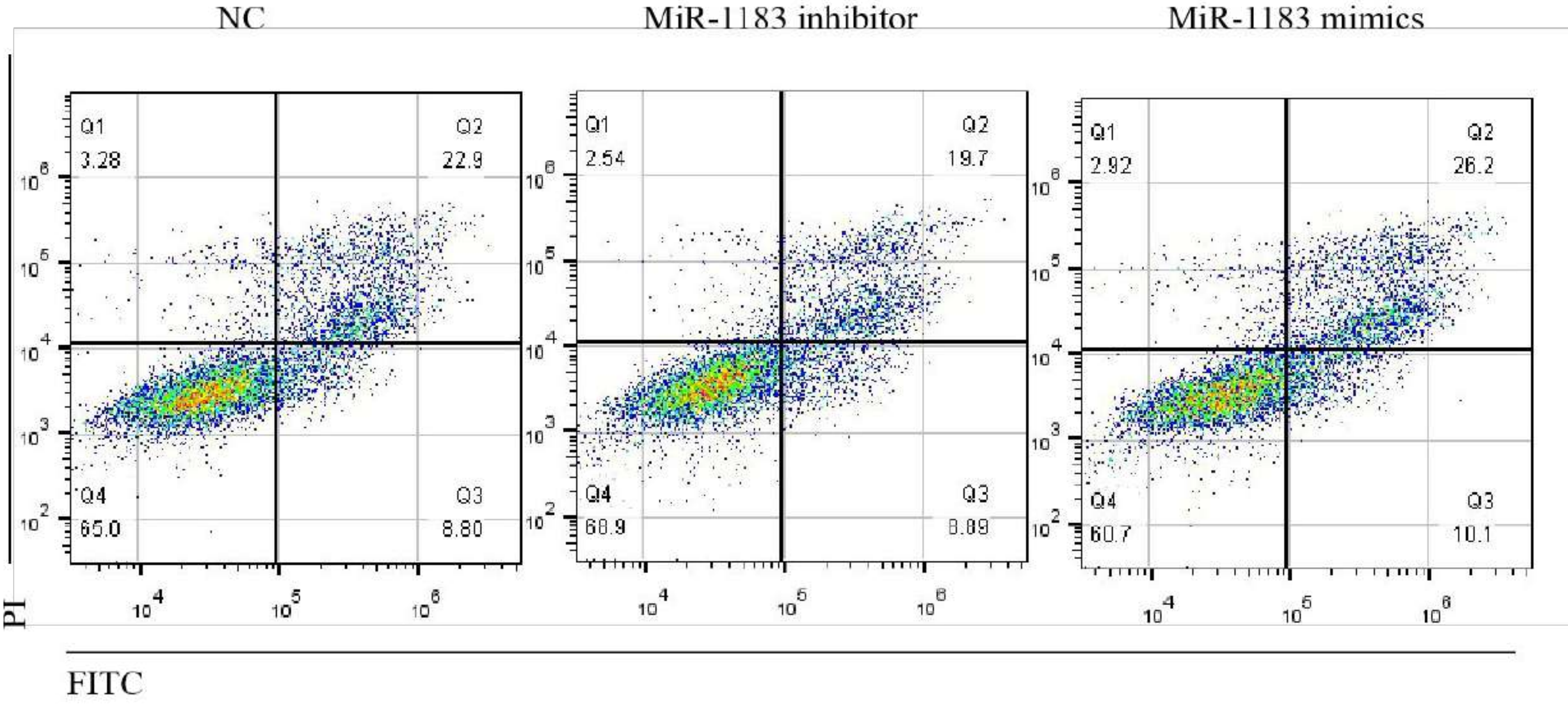
NC
MiR-1183 inhibitor
MiR-1183 mimics
Q1 3.28
Q2 22.9
Q4 65.0
Q3 8.80
Q1 2.54
Q2 19.7
Q4 68.9
Q3 8.89
Q1 2.92
Q2 26.2
Q4 60.7
Q3 10.1
PI
FITC

Supplementary Figure 2. Flow cytometry was used to detect the apoptotic ability on H9C2 cell.

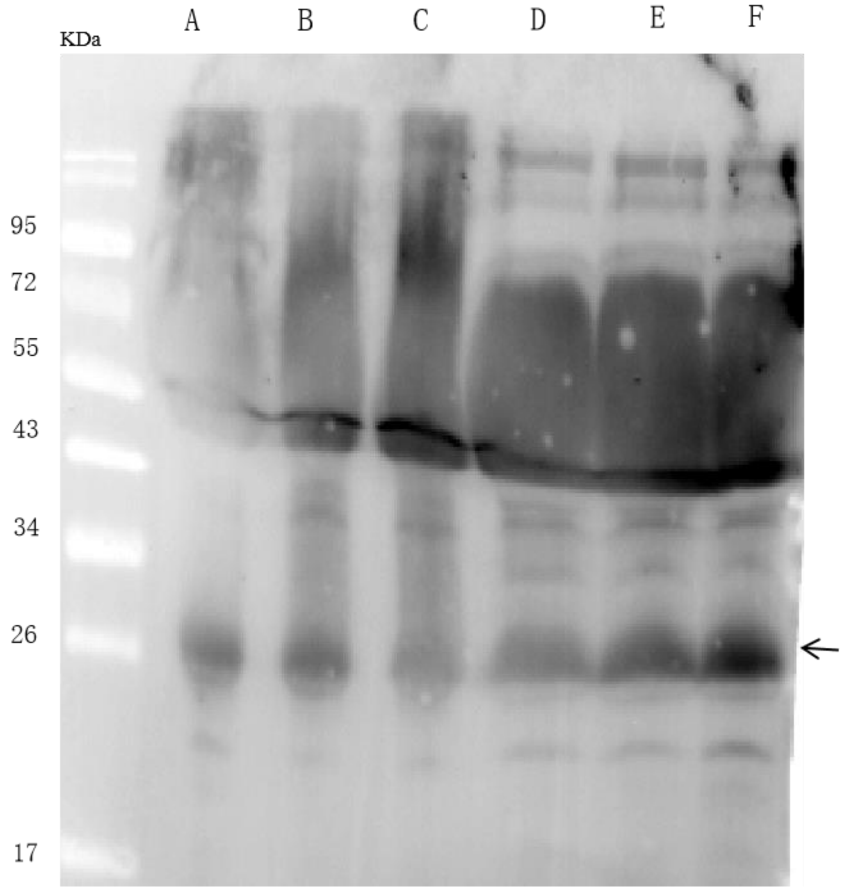

Supplementary Figure 3. Western Blot for the detection the plasma expression of Bcl-2 in RHD patients with high expression of mir-1183.A-C: 3 cases of RHD groups;D-F: 3 control groups.
